# The variant N363S of glucocorticoid receptor in steroid-induced ocular hypertension in Hungarian patients treated with photorefractive keratectomy

**Published:** 2007-04-27

**Authors:** Viktória Szabó, Gábor Borgulya, Tamás Filkorn, Judit Majnik, Ilona Bányász, Zoltán Zsolt Nagy

**Affiliations:** 1 Department of Ophthalmology, Semmelweis University Faculty of Medicine, Budapest, Hungary; 2 Department of Biophysics, KFKI Research Institute for Particle and Nuclear Physics of the Hungarian Academy of Sciences, Semmelweis University Faculty of Medicine, Budapest, Hungary; 3 2nd Department of Medicine, Semmelweis University Faculty of Medicine, Budapest, Hungary; 4 1st Department of Pediatrics, Semmelweis University Faculty of Medicine, Budapest, Hungary

## Abstract

**Purpose:**

Variation in sensitivity to glucocorticoids observed in healthy population is influenced by genetic polymorphisms of the glucocorticoid receptor gene (*NR3C1*). N363S, ER22/23EK, and Bcl I have been previously described as glucocorticoid-sensitivity modulating polymorphisms. We investigated whether these variants may contribute to steroid-induced ocular hypertension and if they play a role as protective or risk factors during exogenous glucocorticoid administration.

**Methods:**

We examined 102 patients who underwent photorefractive keratectomy and received topical steroids (either fluorometholone 0.1% or prednisolone acetate 0.5% alone or combined) as part of postoperative therapy. The choice of steroid depended on course of wound healing and regression. Variations in intraocular pressure (IOP) levels in response to steroid therapy were observed. To genotype DNA, allele-specific PCR amplification was applied for the N363S polymorphism, and PCR-based restriction fragment length polymorphism analysis was performed to examine the Bcl I and the ER22/23EK polymorphisms. We separately analyzed data from three groups of patients: those who received fluorometholone only; those who were initially given fluorometholone then later switched to prednisolone acetate; and those who received prednisolone acetate only. Covariance analysis with forward stepwise variable selection was carried out.

**Results:**

In cases where prednisolone acetate was administered, we found a significant correlation between N363S heterozygosity and steroid-induced ocular hypertension. ER22/23EK and Bcl I polymorphisms do not have a major influence on the risk of developing steroid-induced ocular hypertension.

**Conclusions:**

Genotyping of high risk steroid responders may allow an individual therapy to avoid steroid-induced ocular hypertension. The N363S polymorphism may have a clinical significance in the future.

## Introduction

Photorefractive keratectomy (PRK) is commonly used to correct refractive error of cornea. Postoperative therapy routinely consists of administration of steroid eye drops, because glucocorticoids (GCs) have a dehydrating effect in the corneal stroma. GCs help maintain refractive stability and inhibits stromal scarring, rethickening, and consequent regression of refraction [1-4] although their molecular mechanism is not clearly known [5]. Topical or systemic administration of GCs may produce a rise in intraocular pressure (IOP), but there is considerable variation in rate of occurrence and side effect severity among individuals with GC sensitivity.

GC responsiveness occurs in 10-40% of the normal population, and steroid responders are reported to be at increased risk of developing primary open angle glaucoma as compared with nonresponders [6]. In other clinical studies, occurrence of ocular hypertension in healthy individuals was about 7% in case of four-week long administration of fluorometholone and 35-40% in case of four-week long administration of dexamethasone or betamethasone [2,7]. Similar to dexamethasone, prednisolone acetate has a high propensity to cause ocular hypertension in humans [8].

There is a considerable body of evidence to suggest a role for GCs in the pathogenesis of IOP elevation. GC-induced cellular and morphologic changes in trabecular meshwork may affect resistance to aqueous humor outflow and lead to ocular hypertension [9].

In human eyes, GC and mineralocorticoid receptors were identified in multiple types of tissues. Expression of mRNA of GC receptor was high in the trabecular meshwork and lens epithelium given the effects of GCs in elevated IOP and their ability to induce cataract. Expression of mRNAs encoding mineralocorticoid receptor was high in nonpigmented ciliary epithelium, corneal epithelium, and endothelium [9,10]. Each of the major cell types of the cornea produces glucocorticoid receptor (GR) [5].

It is presumed that the means by which GCs may lead to elevated intraocular pressure is by induction of molecular changes in the trabecular meshwork, resulting in morphologic changes and a reduction in aqueous outflow. These changes include alterations in extracellular matrix production, cell size, nuclear size, DNA content, cytoskeletal organization, phagocytic activity, and protease activity [6,8,11,12].

GC hormones regulate signal transduction in immune and inflammatory systems as well as in growth and development. GCs exert their effects by binding to and activating GC and mineralocorticoid receptors (GR encoded by nuclear subfamily 3, group C, number 1, NR3C1, GeneID; 2908), which are present also in ocular tissues.

GR has a series of discrete domains that mediate the receptor functions of hormone-binding, DNA-binding, and transcriptional modulation [13]. The ligand-receptor complex migrates into the nucleus and binds to the specific DNA sequences called GC response elements (GRE) as a dimer, altering the transcription of target genes, which leads to altered synthesis of proteins. The N363S polymorphism of exon 2 (rs6195) resulting in an asparagine-to-serine change at codon 363 was associated with an increased sensitivity to glucocorticoids. The molecular mechanism of the effect of N363S polymorphism is unknown. However, some authors hypothesized that a putative new phosphorylation site may alter the protein interactions with transcription cofactors [14-17]. The Bcl I restriction site polymorphism was identified as a C/G substitution in intron 2 (IVS2+647), and the G allele was associated with hypersensitivity to GCs [16-20]. The exonic ER22/23EK polymorphism (rs6189, rs6190) consists of a synonymous and a nonsynonymous polymorphism. In codon 22 the nucleotide sequence GAG changes to GAA, both coding for glutamic acid (E). In codon 23 the nucleotide triplet AGG changes to AAG resulting in an amino acid change from arginine (R) to lysine (K) [14]. Previous studies demonstrated ER22/23EK had an association with a hyposensitivity or a relative resistance to GCs [17,21-25]. The allele frequencies in the Caucasian race of the examined polymorphisms were retrieved from the SNPView Database of Ensembl Genome Browser and from recent articles [16,20], and are summerized in Table 1.

**Table 1:** Allele frequencies found in our study population and allele frequencies found in the Caucasian population.

**Polymorphism**	**Genotype**	**Number of patients**	**Allele**	**Allele frequency**	**Allele frequencies in Caucasian population**
N363S	WT:AA	94	A	96.1% (196/204)	91,7-97,9%*
	P:AG	8	G	3.9% (8/204)	2,1%-8,3%*
	P:GG	0			
Bcl I	WT:GG	49	G	71.1% (145/204)	71,1-73%**
	P:GC	47	C	28.9% (59/204)	27-28,9%**
	P:CC	6			
ER22/23EK	WT:GG	99	G	98.5%(201/204)	97-98,7%*
	P:GC	3	C	1.5% (3/204)	1,3-3%*
	P:CC	0			

The genotypic distributions in our study and in literature are shown. Allele frequencies found in our study population are in line with allele frequencies found previously in Caucasian population. Homozygotes carrying the polymorphic alleles were not found in our study population either in the case of N363S polymorphism nor in the case of ER22/23EK polymorphisms. The asterisk indicates the data was taken from the Ensembl Genome Browser SNPView Database. The double asterisk indicates the data were taken from references [16] and [20].

Heterogeneity in responsiveness to GCs may have therapeutic consequences. It may help the choice of the optimal type and dosage of steroids: steroid of low or high efficacy, or steroid administration in combination with antiglaucoma drops.

In our study, we examined a group of patients who underwent treatment with excimer laser PRK and received topical steroids as normal postoperative therapy. The decision to administer either fluorometholone 0.1% or prednisolone acetate 0.5%, or both, was made based on wound healing response and regression of refraction. We investigated whether two exonic polymorphisms (N363S and ER22/23EK) and one intronic variant (Bcl I) of the GR gene were associated with ocular hypertension after topical administration of exogenous GCs.

## Methods

### Patient selection

Caucasian patients (n=102) were recruited from the excimer laser outpatient clinic at the Department of Ophthalmology, Semmelweis University Budapest, Hungary. Each patient was asked to fill out a detailed questionnaire about medical history and current medications. Individuals with endocrine diseases, including diabetes mellitus, were excluded from the study. Participating in the study were 45 women and 57 men. The average age was 35 years (range 21-60 years). The mean preoperative IOP was 16.3 mmHg (range 10-21 mmHg). All patients underwent PRK and received postoperative topical steroids, and gave written consent to participate in the study.

The following selection criteria were used: (1) preoperative IOP less than or equal to 21 mmHg measured by Goldmann applanation tonometry; (2) no personal or family history of glaucoma; (3) at least two follow-up measurements of IOP, the first measurement later than the 21st day, the last one between 5±1 months after the onset of steroid therapy; and (4) continuous steroid administration until the last studied IOP measurement. The same criteria were applied for all analyses.

The study was approved by the ethical committee of Ministry of Welfare and met the requirements of the Declaration of Helsinki.

### Clinical data analysis

All participants underwent preoperative assessment, which included uncorrected and best corrected visual acuity, corneal topography (TMS-1; Tomey, Cambridge, MA), slitlamp biomicroscopy, IOP measurement with Goldmann applanation tonometry, ultrasound pachymetry (Humphrey Model 855; Carl Zeiss, Oberkochen, Germany), retinoscopy following cycloplegia, and fundus examination. The goal of the operation was to gain emmetropy. Prior to PRK refractive surgery, patients were informed of anticipated refractive changes and possible consequences. Informed consent was obtained from each patient before the procedure. PRK was performed with an Aesculap Meditec Mel 70 and 80, 193 nm argon-fluorid excimer laser (Carl Zeiss, Jena, Germany). Each patient was followed up one and five days postoperatively. Reepithelization of the cornea was normal in every case. Patients received topical tobramycin 0.3% five times a day for the first seven days; thereafter, topical fluorometholone 0.1% was started five times daily and continued for six to eight months, in a gradually tapered dose. Patients came every four to six weeks for follow-up visits depending on their status and compliance. In some cases, postoperative therapy was changed to prednisolone acetate 0.5% based on wound-healing response, subepithelial haze, and regression of refraction. Topical prednisolone acetate administration was started if haze graded 1.5 or higher according to Hanna's scale [26] and refractive regression was more than -1.25 D.

To determine whether fluorometholone 0.1% and prednisolone acetate 0.5% raised IOP during postoperative treatment, we divided our study population into three groups: (1) patients treated with fluorometholone only (analysis A1, 132 eyes of 73 patients); (2) patients initially treated with fluorometholone but later switched to prednisolone acetate (analysis A2, 42 eyes of 26 patients); and (3) patients treated with prednisolone acetate only (analysis B, 76 eyes of 38 patients). In analysis A1 and A2, follow-up of the patients started with the date of the initial steroid administration, while in analysis B the follow-up started with the date of switch to prednisolone acetate administration. After IOP elevation was observed, a pressure-lowering therapy was started usually with administration of timolol maleate 0.5%. The parts of the measurement time series after initiation of IOP-lowering therapies were not included in the statistical analysis.

### DNA isolation

Peripheral blood was collected in EDTA-K3 tubes and DNA was isolated from leukocytes using the Wizard DNA Isolation Kit (Promega, Madison, WI).

### Allele-specific PCR for N363S polymorphism

**Figure 1:**
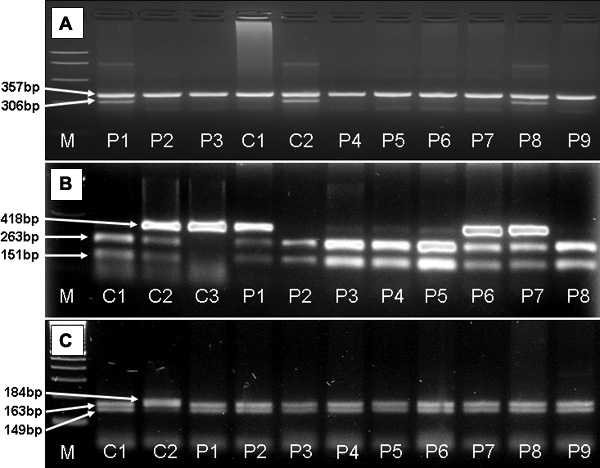
Electrophoresis of products of allele-specific PCR for N363S and PCR-RFLP of Bcl I and ER22/23EK polymorphisms. **A**: Allele-specific PCR reaction yielded a control fragment of 357 bp in each tube and a specific fragment of 306 bp in those tubes where the mutant allele (coding serine) was present. C1 indicates a wild-type homozygous control. C2 indicates a heterozygous control sample. P1 and P8 are patients carrying the polymorphism N363S. P2-P7 and P9 are samples of wild-type patients. A band of 357 bp served as internal control in each reaction. **B**: RFLP analysis using *Bcl*I restriction enzyme resulted in fragments of 263, 151 bp for wild-type homozygous samples (C1, P2-P5, P8), fragments of 418, 263 and 151 bp for heterozygous samples (C2, P1, P6-P7), and a 418-bp-long fragment for polymorphic homozygotes (C3). **C**: Analysis of ER22/23EK using *Mnl*I restriction enzyme resulted in fragments of 149 and 163 bp for wild-type homozygous samples (C1, P1-P9), and fragments of 163 and 184 bp for heterozygous samples (C2). Abbreviations: M=molecular weight marker, bp=base pair, C1-3=control, P1-P9=patients.

Allele-specific PCR amplification was performed with the method designed by Majnik et al. [27] based on the previously described oligonucleotide primers by Koper et al. [14]. Allele-specific PCR reaction yielded a control fragment of 357 bp in each tube and a specific fragment of 306 bp in those tubes where the wild type allele (coding asparagine) or the mutant allele (coding serine) corresponding to the applied specific primer was present. Fragments were separated by agarose gel (3%) electrophoresis, and visualized by ethidium bromide staining (Figure 1A). We used N363N homozygous and N363S heterozygous control DNA samples verified by direct sequencing.

### PCR-based restriction fragment length polymorphism for intronic *Bcl*I polymorphism

DNA was amplified using a standard PCR technique. The forward primer (5'-GAG AAA TTC ACC CCT ACC AAC-3') and the reverse primer (5'-AGA GCC CTA TTC TTC AAA CTG-3') were used at an annealing temperature of 56 °C. The reaction mixture was digested with *Bcl*I restriction enzyme (New England Biolabs, Beverly, MA) at 50 °C for 10 h. The amplified fragment is 418 bp long from which the restriction reaction yielded fragments of 263 and 151 bp for wild-type homozygous samples, fragments of 418, 263, and 151 bp for heterozygous samples, and a 418 bp fragment, which was detected in polymorphic homozygotes. The fragments were separated by agarose gel (2%) electrophoresis, stained with ethidium bromide, and documented using ultraviolet illumination (Figure 1B). Wild-type heterozygous and polymorphic homozygous samples, which had been verified by direct DNA sequencing, were used as internal controls.

### PCR-based restriction fragment length polymorphism for exonic ER22/23EK polymorphism

The forward primer (5'-TGC ATT CGG AGT TAA CTA AAA G-3') and reverse primer (5'-ATC CCA GGT CAT TTC CCA TC-3') were used with a standard PCR protocol; the annealing temperature was 56 °C. The amplified fragment was 448 bp long and was digested with *Mnl*I restriction enzyme (New England Biolabs) at 37 °C for 4 h. This yielded fragments of 149 and 163 bp (and smaller fragments of 50, 49, and 35 bp) in the presence of the wild-type allele and fragments of 163 and 184 bp (and smaller fragments of 50 and 49 bp) in the presence of the polymorphic allele. The fragments were separated by agarose gel (3%) electrophoresis, stained with ethidium bromide, and documented using ultraviolet illumination (Figure 1C). Wild-type homozygous and heterozygous samples were sequenced and used as internal controls.

### Statistical analyses

Analysis of covariance was used with forward stepwise variable selection model biulding. Analysis was carried out with the STATISTICA 5.0 software package. Maximal IOP during follow-up was studied as the endpoint of the analysis; N363S, Bcl I and ER22/23EK were the predictors studied; to adjust for possible confounders ablation depth and pre-treatment IOP were included into the model. Analysis B accounted for a possible further confounder, i.e. duration of fluorometholone therapy. As the left eye:right eye correlation of the maximal IOP values in patients with both eyes treated was higher than 0.9, both eyes of these patients were included in the analysis with weights of 0.5. Patients with only one eye treated were included with a weight of 1.0.

### Photoablation influencing Goldmann applanation tonometry

Some authors have expressed concern about the accuracy of Goldmann applanation tonometry after refractive surgery, as inconsistent results and different proposed correction factors were also published. Excimer laser photoablation alters corneal thickness and rigidity, which thus influences Goldman IOP measurements. Although photoablation depth did not appear as a significant covariant in the regression model, we decided to exclude the role of this possible confounder by comparing the ablation depths of the N363S wild type and heterozygous groups. The photoablation depths of the two groups were almost identical (wild-type group, mean=84.3, SD=23.5; heterozygous group, mean=85.2, SD=27.1).

### Choice of boundaries of follow-up period

In the pilot study we did prior to the present study, we found no elevated IOPs during the first three weeks of follow-up. As a result, we chose to ignore IOP measurements before the 21st day in this study. We chose 5±1 months as the last measurement of the follow-up because we found elevated IOPs were most often observed later than three months, but a large number of patients did not receive steroid treatment for a period longer than six months.

### Normal/elevated intraocular pressure versus intraocular pressure alteration

In clinical practice the diagnosis of elevated IOP is established by comparison to a threshold value (upper boundary of the normal range). In our analyses we did not use thresholds; the IOP measured in mmHg was directly compared to genetic and other factors. This allowed for detection of finer correlations.

## Results

Median duration of steroid administration after PRK was 7.3 months (range: 2.8-30.0 months; 1st quartile/3rd quartile: 5.8/9.2 months). A switch from fluorometholone 0.1% to prednisolone acetate 0.5% therapy was necessary in 37% of the patients after a median fluorometholone 0.1% treatment period of three months (1st quartile/3rd quartile: 1.3/6.0 months). Median photoablation depth was 84 μm (range 13-118 μm; 1st quartile/3rd quartile: 60 μm/99 μm).

The results of the genotyping are summarized in Table 1. Homozygotes carrying the polymorphic alleles were found neither in the case of the N363S polymorphism nor in the case of the ER22/23EK polymorphism.

### Results of analyses A1 and A2

The mean highest IOP during the steroid administration was in analysis A1 and A2 18.0 mmHg (range: 11-36 mmHg; 1st quartile/median/3rd quartile: 16 mmHg/17 mmHg/19 mmHg), 24.8 mmHg (range: 14-44 mmHg; 1st quartile/median/3rd quartile: 19 mmHg/23 mmHg/30 mmHg), respectively. The following variables were included in the model in the forward stepwise selection procedure: N363S, Bcl I, ER22/23EK, photoablation depth, and initial IOP before steroid treatment. No significant predictors of the maximal IOP during follow-up were identified. The forward stepwise selection procedure led to the null model. Thus we could not prove the effect of any genetic or clinical factors on the maximal IOP in the A1 and A2 analyses (Table 2).

**Table 2:** Maximal IOP values in patients carrying various alleles of N363S, Bcl I, and ER22/23EK polymorphisms.

**SNP**	**Genotype**	**Maximal IOP during steroid therapy (mmHg, mean±SD)**		
		**Analysis A1**	**Analysis A2**	**Analysis B**
N363S	WT:AA	18.2±4.3 (n=124)	24.4±8.1 (n=37)	23.4±7.9 (n=70)*
	P:AG	16.0±1.2 (n=8)	27.7±6.2 (n=5)	31.0±7.8 (n=6)*
Bcl I	WT:GG	18.4±4.3 (n=64)	26.7±8.4 (n=23)	25.0±8.9 (n=26)
	P:GC,CC	17.7±4.1 (n=68)	22.5±6.8 (n=19)	23.4±7.3 (n=40)
ER22/23EK	WT:GG	18.0±4.3 (n=131)	24.5±8.0 (n=39)	24.0±8.2 (n=72)
	P:GC,CC	19.0 (n=1)	27.8±6.0 (n=3)	26.3±4.9 (n=4)

Patients carrying the G allele of the N363S polymorphism had significantly higher maximal IOP during the prednisolone acetate treatment (marked with asterisks, p=0.03). Other factors, like frequency of Bcl I and ER22/23EK polymorphisms, photoablation depth, and initial IOP, were statistically significant neither in analysis groups A1 and A2 nor in analysis group B. Abbreviations: SNP: Single nucleotide polymorphism, WT: wild type, P: polymorphic genotype, n: number of eyes studied.

### Results of analysis B

Follow-up started at the first usage of prednisolone acetate 0.5%. Most of the patients were pretreated with fluorometholone 0.1% for various durations. The mean highest IOP during the prednisolone acetate 0.5% therapy was 26.6 mmHg (range: 14-44 mmHg; 1st quartile/median/3rd quartile: 22 mmHg/24 mmHg/34 mmHg). The following variables were included in the forward stepwise variable selection: N363S, Bcl I, ER22/23EK, duration of fluorometholone 0.1% therapy before prednisolone acetate 0.5% administration, photoablation depth, initial IOP before steroid treatment. After the variable selection, only N363S remained in the model, with the coefficient of 9.1 mmHg (SE=4.1 mmHg, p=0.03), which corresponds to a confidence interval of +1.0 to +17.3 mmHg. The other variables did not have a significant effect. Thus we found significant relationship between the N363S heterozygous genotype and higher IOP during prednisolone acetate treatment (Table 2). Residual analysis showed only minor deviation from the normal distribution. This can not substantially deteriorate the accuracy of the results of the model because ANOVA is known to be robust against deviations from normality.

## Discussion

### Genetic background of glucocorticoid-induced ocular hypertension

GCs are widely used in clinical practice. It is well known that some patients develop side effects, even on low dose of GCs, whereas others need a high dose to achieve desired results and do not experience any side effects. In ophthalmology, steroid responsiveness was first described in the early 1960s. GC-induced ocular hypertension was observed by Armaly [7] after topical treatment of the eye both in healthy persons with normal IOP and in patients with glaucoma. Differences in ocular response to steroids in humans was believed to be genetically linked [7,28,29].

### Intraocular pressure response to steroid drugs of different efficacy

GCs, such as fluorometholone and prednisolone acetate, normally inhibit wound healing and are capable of decreasing corneal edema. Therefore, these drugs are administered during the postoperative period after excimer laser surgery. Fluorometholone and prednisolone acetate are steroid drugs of different efficacy. Fluorometholone did not raise IOP as much as dexamethasone and prednisolone acetate, which have a higher propensity to cause ocular hypertension. According to clinical observations, fluorometholone 0.1% caused a 6.1±1.4 mmHg (mean±SD) rise, whereas prednisolone acetate 1.0% caused a 10.0±1.7 mmHg (mean±SD) IOP rise [30,31]. In other clinical study on steroid-responders, a six-week-long treatment with fluorometholone 0.5% produced smaller increases in IOP than did dexamethasone 0.1% [32]. In the study of Leibowitz et al., in the prednisolone-treated group, mean IOP increased significantly from 18.1 to 27.1 mmHg [33].

Therefore, we used three groups to examine occurrence of steroid-induced ocular hypertension after fluorometholone and prednisolone acetate treatment. When we analyzed the association of genotypes and steroid-induced ocular hypertension, we found patients who carryied the N363S polymorphism showed significantly higher IOP during prednisolone acetate treatment. IOP is influenced by multiple factors, presumably the interaction of haplotype and environmental effects. Accordingly, in our study clinically significant IOP elevations requiring topical treatment occurred in patients of the wild-type group as well.

### Functional consequences of glucocorticoid receptor polymorphisms

Previous studies on polymorphisms of the GR gene examined how changes in nucleotide sequence could result in changes in receptor function and in diverse clinical manifestations. Most of the mutations were found to be associated with GC resistance syndromes. However the N363S variant and *Bcl*I restriction site polymorphism resulted in GC hypersensitivity; the ER22/23EK polymorphism was associated with relative resistance to GCs.

There are at least 16 GR isoforms in a cell, combined with several phosphorylation sites, therefore GR presents an enormous potential for signaling diversity [34,35]. Various parameters determine GC responsiveness, and several proteins interact with the GR. The mechanism of GC sensitivity may be elucidated by studies on GR-interacting proteins. Transcriptional activation by GR of target genes is subsequent to ligand binding, conformational changes, contact with chaperones, transcription factors, and other proteins [36]. Polymorphisms in GR may change the function of GR-interacting proteins, which may influence sensitivity to GCs. These mechanisms may explain variation in responsiveness to GC and GC-induced side effects. The ER22/23EK polymorphism resulted in a significant reduction of transactivating capacity. The N363S polymorphism resulted in a significantly higher transactivating capacity. However, neither the ER22/23EK nor the N363S polymorphism seemed to influence the transrepressing capacity of the GR [22].

### Maximal intraocular pressure versus intraocular pressure alteration

As we evaluated data from this study, we considered whether to take the maximal IOP during follow-up as the dependent variable of the statistical analysis or the individual IOP alteration (maximal IOP minus preoperative IOP). Both analyses were evaluated, which led to a decisive result. When treating IOP alteration as the target of the analysis, the preoperative IOP was a significant covariate with a coefficient of -1.02. As this coefficient was close to -1.0, we concluded it was futile to use the preoperative IOP a predictor of maximal IOP during steroid treatment. Thus, our analysing method of choice targeted the maximal IOP. So the studied factors predict the maximal IOP independently of the preoperative IOP. Although this finding enabled us to carry out a simpler analysis, we do not recommend this analysis method in general. Certain diseases may cause some patients to react in unpredictably.

### Effect of previous fluorometholone treatment

We thought about the fluorometholone 0.1% treatment preceding the prednisolone acetate 0.5% treatment to be the better predictor of steroid response, so we studied its effect in an additional statistical analysis as well. We forced the duration of previous fluorometholone 0.1% treatment variable into the model, excluding it from the stepwise variable selection. The parameter estimate corresponding to previous treatment duration was minor and nonsignificant. Thus the effect of the previous fluorometholone 0.1% treatment seems to be insignificant compared to the effect of prednisolone acetate 0.5% treatment.

In summary, GCs are drugs commonly used to treat systemic diseases, such as immune diseases, chronic inflammation, or to prevent of rejection of organ grafts. High-dose oral GCs may cause side effects, affecting various organs to different extents. Each subject, when treated with GCs, needs an individually optimized dose to maintain a balance between beneficial and adverse effects. By using genotype to identify high-risk steroid responders may allow for individualized therapy, enabling the avoidance of GC side effects. In order to optimize individual GC treatments, N363S genotyping may become everyday practice.

In our study of a modest number of cases, we showed that the N363S polymorphism of the GC receptor gene was significantly associated with increased IOP as response to prednisolone acetate 0.5% therapy after PRK. In the future, N363S genotyping before steroid treatment may have a role in preventing steroid-induced high IOP.
